# Risk factors for miscarriage in Syrian refugee women living in non-camp settings in Jordan: results from the Women ASPIRE cross-sectional study

**DOI:** 10.1186/s13031-022-00464-y

**Published:** 2022-06-07

**Authors:** Maysa M. Khadra, Haya H. Suradi, Justin Z. Amarin, Nabila El-Bassel, Neeraj Kaushal, Ruba M. Jaber, Raeda Al-Qutob, Anindita Dasgupta

**Affiliations:** 1 Department of Obstetrics and Gynecology, The University of Jordan School of Medicine, Queen Rania Street, Amman, 11942 Jordan; 2 The University of Jordan School of Medicine, Amman, Jordan; 3 Columbia University School of Social Work, New York City, NY USA; 4 Department of Family and Community Medicine, The University of Jordan School of Medicine, Amman, Jordan

**Keywords:** Maternal health, Miscarriage, Pregnancy outcome, Pregnant women, Refugees, Risk factors, Syria

## Abstract

**Background:**

Syrian refugee women face health care disparities and experience worse pregnancy outcomes, including miscarriage. We investigated risk factors for miscarriage in Syrian refugee women living in non-camp settings in Jordan to identify targets for interventions.

**Methods:**

We analyzed data from Women ASPIRE, a cross-sectional study of gendered physical and mental health concerns of 507 Syrian refugee women (≥ 18 years old) living in non-camp settings in Jordan. We recruited women using systematic clinic-based sampling from four clinics. We limited our analyses to women who had a history of pregnancy and whose most recent pregnancy was single, took place in Jordan, and ended in term live birth or miscarriage (*N* = 307). We grouped the women by the primary outcome (term live birth or miscarriage) and compared the sociodemographic and clinical characteristics of the two groups. We used Pearson’s *χ*^2^ test or the Mann–Whitney *U* test to obtain unadjusted estimates and multivariable binomial logistic regression to obtain adjusted estimates.

**Results:**

The most recent pregnancies of 262 women (85%) ended in term live birth and another 45 (15%) ended in miscarriage. Since crossing into Jordan, 11 women (4%) had not received reproductive health services. Of 35 women who were ≥ 35 years old, not pregnant, and did not want a (or another) child, nine (26%) did not use contraception. Of nine women who were ≥ 35 years old and pregnant, seven (78%) did not plan the pregnancy. The adjusted odds of miscarriage were higher in women who had been diagnosed with thyroid disease (aOR, 5.54; 95% CI, 1.56–19.07), had been of advanced maternal age (aOR, 5.83; 95% CI, 2.02–16.91), and had not received prenatal care (aOR, 36.33; 95% CI, 12.04–129.71). Each additional previous miscarriage predicted an increase in the adjusted odds of miscarriage by a factor of 1.94 (1.22–3.09).

**Conclusions:**

We identified several risk factors for miscarriage in Syrian refugee women living in non-camp settings in Jordan. The risk factors may be amenable to preconception and prenatal care.

## Background

Since the beginning of the Syrian Civil War, millions of people have been internally displaced, and millions more have become refugees. Over 5.6 million Syrian refugees are registered with the UNHCR [1]. Countries neighboring Syria host a large proportion of Syrian refugees; according to UNHCR, approximately 661,997 Syrian refugees reside in Jordan, and estimates from the national Ministry of Health are closer to one million [1, 2]. Relative to the host community, Syrian refugees in Jordan bear a high burden of both communicable and noncommunicable diseases, such as influenza and hypertension, likely due to poor access to and low utilization of health care services [3, 4]. In addition to high rates of communicable and noncommunicable disease, and higher rates of mental illness relative to the host community, Syrian refugee women also contend with disproportionately high rates of poor maternal and child health outcomes, such as spontaneous abortion (noninduced embryonic or fetal death or passage of products of conception before 20 weeks gestation)—commonly referred to as “miscarriage” [5–7]. These health disparities are hypothesized to be due, at least in part, to poor access to health services among Syrian refugee women in Jordan [5].

Globally, 12–15% of pregnancies end in miscarriage [8, 9], however research highlights higher rates of miscarriage among refugee populations compared to host communities. Torun et al*.* sampled Syrian refugee women living in İstanbul, Turkey and found that 23.1% of pregnancies ended in miscarriage [10]. Harakow et al*.* performed a systematic review of pregnancy complications in refugee women and found an increased risk of miscarriage among refugee women compared to women from host populations [11]. The critical need to address this disparity is further heightened due to the high proportion of maternal mortality often associated with miscarriage; Hynes et al*.* studied maternal deaths that occurred in 25 refugee camps in 10 countries, and found that 78% of maternal deaths followed miscarriage or complicated delivery [12].

While the literature cited above highlights novel research documenting elevated rates of miscarriage among refugee populations relative to host communities, little research has been conducted to understand risk factors for miscarriage. Research among general populations of women indicate that chromosomal abberations, which increase with advanced maternal age are primary drivers of miscarriage [13, 14]. In addition, elevated rates of miscarriage have also been linked to chronic diseases including thyroid diseases or diabetes mellitus, gendered inequities of health such as domestic violence, and mental illness like depression, and posttraumatic stress disorder [15–19]. While these conditions are caused by a variety of environmental factors, there are some related modifiable risk factors that may be intervened on to potentially reduce the risk of miscarriages—such as engaging in prenatal care [14].

Prenatal care present opportunities to modify risk factors for miscarriage. In most cases, miscarriage is irreversible, which makes prevention the most effective intervention [14]. During prenatal care appointments, there is an opportunity for screening and providing support for many health conditions such as chronic disease (medications), domestic violence (counseling, safety planning, engagement in social services), and mental illness (medication and counseling) [19, 20]. Unfortunately, numerous studies have shown that the prenatal care coverage of Syrian refugee women is inadequate. According to a population-based study of Syrian refugees in Jordan, 18% of women and girls whose most recent pregnancy was in Jordan had not received prenatal care [4]. Benage et al*.* reported that 17.1% of 420 pregnant Syrian refugee women in Lebanon had not received prenatal care [21]. In Turkey, Şimşek et al*.* interviewed 458 Syrian refugee women living in non-camp settings and found that 26.7% had not received prenatal care [7]. In addition, the needs of Syrian refugee women who do report for preconception and prenatal care may not be met [22]. However, little research has been conducted in Jordan to identify factors associated with increased odds of miscarriage in Syrian refugee women. We sought to build on existing literature to identify risk factors for miscarriage in a sample of health care–seeking Syrian refugee women in Jordan. We hypothesized that a combination of sociodemographic, physical and mental illness, and gendered inequities of health will be associated with increased of miscarriage in this sample.

## Method

### Design and setting

We analyzed data from Women ASPIRE, a cross-sectional study of gendered physical and mental health concerns of Syrian refugee women living in non-camp settings in Jordan (*N* = 507). The key objective of this study was to understand which health concerns are most prevalent for Syrian refugee women who were engaged in health care systems, as well as modifiable risk factors to reduce negative health outcomes in order to help inform best practices in clinical, and policy settings to improve the lives of Syrian refugee women in Jordan. Additional details on the Women ASPIRE study have been published elsewhere [19, 23]. We limited our analyses to women who had a history of pregnancy and whose last pregnancy (which would have taken place in the year prior to data collection, 2017) was single, took place in Jordan, and ended in term live birth or miscarriage (*N* = 307). The study was set in four nongovernmental health clinics that primarily served women and children, one in each of four governorates in Jordan (al-Mafraq, Amman, az-Zarqā’, and Irbid), which hosted the majority of all registered Syrian refugees in Jordan [24]. Research assistants with a degree in medicine, psychology, public health, or sociology approached women in the clinic waiting rooms, introduced the study, obtained informed consent, and enrolled eligible women into the study. Research staff then conducted a one-hour, face-to-face, interviewer-administered quantitative survey using Qualtrics software on electronic tablets. Upon completion of the survey, each participant received a food package (valued at 7 USD). The research assistants completed training in human subjects research, referral pathways, and survey administration.

### Participants

We recruited eligible women by time- and venue-based systematic sampling between April and November 2018. We included women who were Syrian refugees (confirmed by UNHCR identification card), were ≥ 18 years old, lived in non-camp settings in Jordan, were visiting the clinic for their own health, and agreed to participate. We excluded women with any degree of cognitive dysfunction as determined by the Mini–Mental State Examination [25]. Of all women invited to participate, 84% met the eligibility criteria and agreed to participate (*N* = 507).

### Measures

For the present analysis, we aimed to identify factors associated with increased risk of miscarriage among our sample. We selected a series of demographic, physical and mental illnesses, and gendered inequities of health that have been previously found to be associated with miscarriage in comparable samples.

#### Sociodemographic characteristics

We measured age (in years) on a continuous scale, and defined advanced maternal age as age ≥ 35 years at conception [13]. We determined literacy based on the standard definition (ability to read and write). We asked participants whether they were married and, if applicable, the age at first marriage. We then dichotomized the age at first marriage as ≥ 18 years or < 18 years to construct the child marriage variable.

We used the reduced Coping Strategies Index (CSI) to measure household food insecurity (Cronbach’s *ɑ* = 0.67). This measure, which has been validated for use with displaced Arab populations, and was selected to characterize the economic stability of the household (which has been shown to be associated with poor reproductive health outcomes, globally), rather than using a measure such as household income, given that most refugee families received similar financial assistance from various aid organizations. The reduced CSI is a standard set of five coping strategies that indicate household food insecurity [26]. We assessed whether participants had resorted to the five coping strategies within the past 30 days using an individual “Yes” or “No” scale for each strategy. Then, we summed the number of “Yes” responses (0–5) and considered two or more “Yes” responses to indicate food insecurity.

We captured a simplified residential history using two items from the questionnaire: the governorate of permanent residence in Syria (Aleppo, al-Ḥaskah, al-Lādhiqīyah, al-Qunayṭrah, ar-Raqah, as-Suwaydā’, Damascus, Dar ‘ā, Dayr al-Zūr, Ḥamāh, Ḥimṣ, Idlib, Reef Demashq, or Ṭarṭūs) and the governorate of current residence in Jordan (‘Ajlūn, Amman, al-’Aqabah, al-Balqā’, al-Karak, al-Mafraq, aṭ-Ṭafīlah, az-Zarqā’, Irbid, Jarash, M’ān, Mādabā). We collapsed the categories of both variables based on geographical clustering.

#### Domestic physical violence

We measured the lifetime exposure to domestic physical violence, perpetrated by the current or most recent husband (Cronbach’s *ɑ* = 0.75) or a family member (Cronbach’s *ɑ* = 0.68), using a modified version of the Conflict Tactics Scale, which has been validated in a sample of Palestinians [27, 28]. We asked participants to report, using a “Yes” or “No” scale, whether they had experienced eight forms of physical abuse. Women who responded “Yes” to any one of the eight items were considered to have been exposed to physical abuse and those who responded “No” to all eight items were considered not to have been exposed.

#### Physical health characteristics

We asked participants whether they had ever been diagnosed with thyroid disease or diabetes mellitus with binary response options of either “Yes” or “No.” We also asked participants who had been pregnant at least once before about the outcome, order, and prenatal care for up to five pregnancies, in order of recency. To measure the number of previous miscarriages, we enumerated the number of pregnancies based on data we collected on the most recent five pregnancies experienced. We collected data on the five most recent pregnancies because we expected that most women in our sample would have come to Jordan from Syria within the past 5 years. Out of up to the four pregnancies preceding the last pregnancy—that resulted in miscarriage, and retained the variable as a continuous variable.

#### Mental health characteristics

We measured mental health using three validated scales—namely: the abridged Center for Epidemiologic Studies Depression (CES–D) four-item scale, Generalized Anxiety Disorder seven-item (GAD–7) scale, and Posttraumatic Stress Disorder Checklist for DSM–5 (PCL–5). CES–D measures symptoms of depression based on seven-day recall, GAD–7 measures symptoms of generalized anxiety disorder based on two-week recall, and PCL–5 measures symptoms of posttraumatic stress disorder based on 30-day recall [29–31]. We used the standard cut scores (≥ 4, ≥ 10, and ≥ 23, respectively) to interpret the screening results on a dichotomous scale (“negative” or “positive”). Cronbach’s *ɑ* for CES–D, GAD–7, and PCL–5 were 0.78, 0.85, and 0.94, respectively.

#### Dependent variable

The primary dependent variable of this analysis was whether the most recent pregnancy ended in term live birth or miscarriage. We created a dichotomous variable based on the question “What was the outcome for this pregnancy?” We defined term live birth as the delivery of a neonate with signs of life after 37 weeks of gestation and miscarriage as spontaneous pregnancy loss before 20 weeks of gestation.

### Data analysis

We computed summary statistics to describe the background characteristics and access to health services of the full sample (*N* = 307 women). We then performed a subgroup analysis of women of advanced maternal age to describe the rates of unplanned pregnancies and contraception use. Next, we estimated the relationships between independent variables and the most recent pregnancy outcome (term live birth or miscarriage) for the full sample using Pearson’s *χ*^2^ test or the Mann–Whitney *U* test. Finally, we fit a binary logistic regression model (with Firth’s correction for small-sample bias) to obtain adjusted estimates [32]. We included in the model the full set of independent variables, namely “literacy”, “household food insecurity”, “region of permanent residence in Syria”, “region of current residence in Jordan”, “lifetime diagnosis of diabetes mellitus”, “lifetime diagnosis of thyroid disease”, “awareness of a reproductive health service in the area of residence”, “advanced maternal age at conception”, “prenatal care”, “number of previous miscarriages”, “child marriage”, “lifetime physical abuse by the current or most recent husband”, “lifetime physical abuse by a family member”, “CES–D result”, “GAD–7 result”, and “PCL–5 result”. We excluded eight incomplete cases from the multivariable analysis and we used the Benjamini–Hochberg method to correct for multiple testing. We used R (version 3.6.2) to perform all data analyses.

#### Ethics

The Columbia University Morningside Institutional Review Board and the Jordanian Ministry of Health approved the study protocol. All participants provided written informed consent.

## Results

### Background sample characteristics and health service utlization

The mean age of the women was 29.0 years (SD: 7.0), with similar mean ages for women delivering live births (27.1, SD: 6.1), and those who had miscarriages (27.8; SD: 7.4), with a range of 18–48 years. The majority of women had crossed into Jordan in 2011 (12.7%), 2012 (26.4%), 2013 (33.9%), and 2014 (20.8%). In total, 296 women (96.4%) had received reproductive health services since crossing into Jordan and 11 (3.6%) had not. Of the latter, 6 (54.5%) required the services. Of the former, 213 (72.0%) had received the services during the previous six months. The most popular service providers were nongovernmental organizations, private clinics, and the Jordanian Ministry of Health.

### Rates of unplanned pregnancies and contraception use in women of advanced maternal age

At the time of interviews, 67 women (21.8%) were of advanced maternal age. Of those, 22 (32.8%) wanted a (or another) child, 39 (58.2%) did not want a (or another) child, and six (9.0%) were undecided. Of 35 women who were of advanced maternal age, not pregnant, and did not want a (or another) child, 26 (74.3%) used contraception but nine (25.7%) did not. Of nine women who were of advanced maternal age and pregnant, two (22.2%) planned the pregnancy but seven (77.8%) did not. Of 133 women who married as children, 123 (92.5%) were not of advanced maternal age during the most recent pregnancy. On the other hand, of 172 women who were not married as children, 140 (81.4%) were not of advanced maternal age during the most recent pregnancy.

### Risk factors for miscarriage

We outline the sociodemographic and clinical characteristics of the 307 women, stratified by the most recent pregnancy outcome (term live birth or miscarriage), in Table 1. The rate of thyroid disease was 22.2% for women who miscarried compared with 6.1% for those who had a term live birth (*p* = 0.002). In addition, 30.2% of women who miscarried were of advanced maternal age at conception, while 11.1% of those who had a term live birth were of advanced maternal age at conception (*p* = 0.003). Almost all women who had a term live birth (*n* = 256, 97.7%) received prenatal care, while approximately half of those who miscarried (*n* = 24, 54.5%) received prenatal care (*p* < 0.001). Child marriage was almost twice as common in women who had a term live birth (*n* = 121, 46.2%) compared with those who had a miscarriage (*n* = 12, 26.7%; *p* = 0.039). In contrast, lifetime physical abuse by a family member was almost twice as common in women who had a miscarriage (*n* = 17, 37.8%) compared with those who had a term live birth (*n* = 55, 21.0%; *p* = 0.039). Finally, women who miscarried had a median of one previous miscarriage, while women who delivered a term live birth had a median of no previous miscarriages (*p* < 0.001).

**Table 1:** Sociodemographic and clinical characteristics of Syrian refugee women whose last single pregnancy in Jordan resulted in term live birth or miscarriage (*N* = 307)

	Total (*N* = 307)	Term live birth (*n* = 262)	Miscarriage (*n* = 45)	*p* value
Age, mean (SD)	29.0 (7.0)	27.1 (6.1)	27.8 (7.4)	0.57
Literacy				0.40
No	43 (14.0)	39 (14.9)	4 (8.9)	
Yes	264 (86.0)	223 (85.1)	41 (91.1)	
Household food insecurity				0.40
No	88 (28.7)	78 (29.8)	10 (22.2)	
Yes	219 (71.3)	184 (70.2)	35 (77.8)	
Region of permanent residence in Syria				0.47
South Syria	137 (44.6)	114 (43.5)	23 (51.1)	
Central Syria	54 (17.6)	45 (17.2)	9 (20.0)	
North Syria	116 (37.8)	103 (39.3)	13 (28.9)	
Region of current residence in Jordan				0.54
Central Jordan	171 (55.7)	148 (56.5)	23 (51.1)	
North Jordan	136 (44.3)	114 (43.5)	22 (48.9)	
Lifetime diagnosis of diabetes mellitus				0.072
No	288 (93.8)	249 (95.0)	39 (86.7)	
Yes	19 (6.2)	13 (5.0)	6 (13.3)	
Lifetime diagnosis of thyroid disease				0.002
No	281 (91.5)	246 (93.9)	35 (77.8)	
Yes	26 (8.5)	16 (6.1)	10 (22.2)	
Awareness of a reproductive health service in the area of residence				0.40
No	119 (38.8)	98 (37.4)	19 (46.7)	
Yes	188 (61.2)	164 (62.6)	24 (53.3)	
Advanced maternal age at conception				0.003
No	263 (86.2)	233 (88.9)	30 (69.8)	
Yes	42 (13.8)	29 (11.1)	13 (30.2)	
Prenatal care				< 0.001
No	26 (8.5)	6 (2.3)	20 (45.5)	
Yes	280 (91.5)	256 (97.7)	24 (54.5)	
Number of previous miscarriages, median (range)	0 (0–4)	0 (0–4)	1 (0–3)	< 0.001
Child marriage				
No	174 (56.7)	141 (53.8)	33 (73.3)	0.039
Yes	133 (43.3)	121 (46.2)	12 (26.7)	
Lifetime physical abuse by the current or most recent husband				0.40
No	120 (39.7)	106 (40.9)	14 (32.6)	
Yes	182 (60.3)	153 (59.1)	29 (67.4)	
Lifetime physical abuse by a family member				0.039
No	235 (76.5)	207 (79.0)	28 (62.2)	
Yes	72 (23.5)	55 (21.0)	17 (37.8)	
CES–D result				0.70
Negative	117 (38.1)	101 (38.5)	16 (35.6)	
Positive	190 (61.9)	161 (61.5)	29 (64.4)	
GAD–7 result				0.42
Negative	136 (44.3)	119 (45.4)	17 (37.8)	
Positive	171 (55.7)	143 (54.6)	28 (62.2)	
PCL–5 result				0.33
Negative	110 (35.8)	98 (37.4)	12 (26.7)	
Positive	197 (64.2)	164 (62.6)	33 (73.3)	

Categorical data are presented as *n* (%)

### Multivariable analysis

Table 2 summarizes the multivariable analysis conducted to identify associations between demographic, physical and mental illnesses, health care utilization, and gendered inequities of health and the dependent variable of miscarriage. The adjusted odds of miscarriage were higher in women who had been diagnosed with thyroid disease (aOR, 5.54; 95% CI, 1.56–19.07), had been of advanced maternal age (aOR, 5.83; 95% CI, 2.02–16.91), and had not received prenatal care (aOR, 36.33; 95% CI, 12.04–129.71). In addition, each additional previous miscarriage predicted an increase in the adjusted odds of miscarriage by a factor of 1.94 (1.22–3.09). Child marriage and lifetime physical abuse by a family member, which were statistically significantly associated with the outcome in unadjusted analyses, were not statistically significant predictors.

**Table 2:** Factors associated with miscarriage at last pregnancy among Syrian refugee women in Jordan (*N* = 299 women)

	Unadjusted OR (95% CI)	Adjusted OR (95% CI)	*p* value
Literacy			0.38
No	Reference	Reference	
Yes	1.79 (0.61–5.29)	3.24 (0.71–18.17)	
Household food insecurity			
No	Reference	Reference	
Yes	1.48 (0.70–3.14)	0.62 (0.23–1.75)	0.77
Region of permanent residence in Syria			
South Syria	Reference	Reference	
Central Syria	0.63 (0.25–1.58)	0.98 (0.26–3.11)	0.98
North Syria	1.01 (0.43–2.35)	0.66 (0.20–2.10)	0.90
Region of current residence in Jordan			0.93
Central Jordan	Reference	Reference	
North Jordan	1.24 (0.66–2.34)	1.34 (0.46–3.85)	
Lifetime diagnosis of diabetes mellitus			0.93
No	Reference	Reference	
Yes	2.95 (1.06–8.21)	0.85 (0.16–3.88)	
Lifetime diagnosis of thyroid disease			0.039
No	Reference	Reference	
Yes	4.39 (1.85–10.44)	5.54 (1.56–19.07)	
Awareness of a reproductive health service in the area of residence			0.93
No	Reference	Reference	
Yes	0.68 (0.36–1.29)	0.86 (0.36–2.07)	
Advanced maternal age at conception			0.01
No	Reference	Reference	
Yes	3.48 (1.63–7.42)	5.83 (2.02–16.91)	
Prenatal care			< 0.001
No	Reference	Reference	
Yes	0.03 (0.01–0.08)	0.03 (0.01–0.08)	
Number of previous miscarriages	2.15 (1.51–3.05)	1.94 (1.22–3.09)	0.03
Child marriage			0.42
No	Reference	Reference	
Yes	0.42 (0.21–0.86)	0.54 (0.21–1.30)	
Lifetime physical abuse by the current or most recent husband			0.93
No	Reference	Reference	
Yes	1.44 (0.72–2.84)	0.87 (0.30–2.48)	
Lifetime physical abuse by a family member			0.15
No	Reference	Reference	
Yes	2.29 (1.17–4.47)	2.69 (1.03–7.09)	
CES–D result			0.93
Negative	Reference	Reference	
Positive	1.14 (0.59–2.20)	0.90 (0.28–2.87)	
GAD–7 result			0.93
Negative	Reference	Reference	
Positive	1.37 (0.72–2.63)	0.86 (0.25–3.02)	
PCL–5 result			0.93
Negative	Reference	Reference	
Positive	1.64 (0.81–3.33)	0.91 (0.26–3.17)	

## Discussion

We investigated risk factors for miscarriage in Syrian refugee women living in non-camp settings in Jordan and found that women who miscarried were more likely to have a combination of risk factors related to physical health, obstetric history, and gendered inequities. Previous studies have estimated the rate of miscarriage in Syrian refugee women, but none have investigated the risk factors for miscarriage in this population. Therefore, this is the first study to identify specific risk factors for miscarriage in a sample of Syrian refugee women and document the significant need to address these risk factors to reduce rates of miscarriage among Syrian refugee women.

Thyroid diseases and diabetes mellitus are often cited as risk factors for miscarriage [15]. For example, Su et al*.* conducted a prospective, population-based cohort study and found that isolated hyperthyroxinemia was associated with miscarriage [33]. In addition, Zhang et al*.* concluded, from a systematic review and meta-analysis of nine reports, that subclinical hypothyroidism is a risk factor for miscarriage [34]. On the other hand, Kalter reviewed more than 50 reports and concluded that diabetes mellitus is not a risk factor for miscarriage [35]. In support, we found that a lifetime diagnosis of thyroid disease was associated with miscarriage but diabetes mellitus was not. According to a cross-sectional study of 8,041 Syrian refugee adults living in non-camp settings, the prevalence of thyroid diseases and diabetes was 2.5 and 9.2%, respectively. In addition, 25.6 and 13.4% of people with the conditions, respectively, did not seek care when needed [36]. In general, the risk of miscarriage in women with well-controlled thyroid diseases, which affect 2–3% of pregnant women, is no different than the risk of miscarriage in healthy women, and treatment with levothyroxine lowers the risk of miscarriage in women with clinical hypothyroidism and may lower the risk of miscarriage in women with thyroid autoimmunity [37]. Therefore, our results—in tandem with the results of Rehr et al*.*—highlight a target for intervention [36]. In our sample, the higher rate of a lifetime diagnosis of thyroid disease in women who miscarried (22.2% vs. 6.1% suggests poor control of active thyroid disease. We recommend that Syrian refugee women who attend prenatal care should be screened for thyroid disease. Further studies are needed to assess the prevalence of laboratory-proven thyroid dysfunction and appropriate medication use in this population.

Nybo Andersen et al*.* showed that the risk of miscarriage increases with maternal age, independent of the number of previous miscarriages, and our results support the previous [38]. Several factors explain the association of advanced maternal age with miscarriage, including increased likelihood of chromosome aberrations and a decrease in uterine and hormonal function [14, 38]. We also found that most nonpregnant women of advanced maternal age did not want more children. However, a quarter of them did not use contraception. In addition, most women of advanced maternal age who were pregnant at the time of data collection had not planned to become pregnant. It is possible that women who were of advanced maternal age and did not wish to get pregnant may not have wanted to engage as closely with prenatal care and reproductive health care services, as well. According to qualitative data collected from focus groups, Syrian refugee women in Jordan and Lebanon are interested in family planning services but the main barriers are health services inaccessibility and cost [39, 40]. Therefore, increased accessibility to family planning services may limit the number of unwanted pregnancies in women of advanced maternal age and thereby limit the number of miscarriages associated with advanced maternal age.

Chromosome aberrations cause most miscarriages [41]. However, Feodor Nilsson et al*.* estimated that up to 25.2% of miscarriages may be prevented by preconception and prenatal care [14]. Previous studies have shown that pregnant refugee women are more likely to receive substandard prenatal care, which puts them at an increased risk of complications [42]. In Jordan, prenatal care is provided free of cost to refugees at numerous nongovernmental and governmental agencies throughout the country. However, utilization of these services varies throughout the country. We found that almost all of the women whose most recent pregnancy was a term live birth received prenatal care, which may be indicative of prenatal care service availability to refugees in Jordan. On the other hand, almost half of the women whose most recent pregnancy ended in miscarriage did not receive prenatal care, highlighting the critical need to ensure prenatal care utilization to reduce risk of miscarriage. Importantly, most miscarriages occur during the first trimester—perhaps before the first prenatal care visit [43]. Indeed, Tappis et al*.* reported that 44.1% of a comparable sample of Syrian refugee women in Jordan attended the first prenatal care visit during the first trimester [4]. Consideration of prenatal care utilization in our sample is of particular interest given that all study participants were engaged in health care settings by nature of our eligibility criteria. Agencies where this study took place all provided strong prenatal care services free of charge, and were tailored to the needs of Syrian refugee women. However, we still saw low rates of prenatal care among those who miscarried. Future research must focus on implementation research approaches to understand the best way to address barriers to prenatal care in low-resourced settings. In addition, study participants lived in non-camp settings. Health services in camp settings provide a different opportunity to deliver prenatal care services to women, given that health care workers in camp settings may have more opportunities to directly reach out to refugee women due to physical proximity.

We also found that child marriage was associated with term live birth only in the unadjusted analysis. In our sample, the relationship between child marriage and term live birth may be explained by advanced maternal age; we found that most women who married as children were not of advanced maternal age during the most recent pregnancy. Child marriage increases within the context of humanitarian conflicts and disasters, as has been well documented during the Syrian Civil War [44]. Indeed, Ozel et al*.* found that the rate of adolescent pregnancy was higher in Syrian refugee women than in ethnic Turkish women [45]. Notably, early pregnancy increases the risk of many complications but not miscarriage [38, 44]. In addition, we found that lifetime physical abuse by a family member was associated with miscarriage only in the unadjusted analysis as well. In support, Silverman et al*.* showed that peripregnancy violence from in-laws, though not collinear with intimate partner violence, was associated with less uptake of prenatal care [46]. Therefore, lifetime physical abuse by a family member may be a marker of poor prenatal care utilization, and violence perpetrated by family members should be addressed during screens for domestic abuse in the initial prenatal visit or through community outreach efforts.

Our study has several limitations. First, we did not establish the time order of some exposures and the most recent pregnancy outcome, so the associations we found must be interpreted with caution. Second, we did not collect data on important risk factors for miscarriage, such as maternal infections, consanguity, substance use, and environmental exposures, and details on types of prenatal care (ex. number of prenatal care visits, taking prenatal vitamins, etc.), and we were limited in how many variables were able to be included in the final multivariable models based on our overall sample size, and therefore could not include additional variables of interest such as anemia history, or age at first pregnancy. Nevertheless, many of the risk factors we studied are modifiable and account for a significant proportion of preventable miscarriages. Third, some rates, such as those of domestic physical violence, may be underestimated due to social desirability bias. However, we used closed-ended, validated measures to limit social desirability bias. Fourth, our sample may be biased because we recruited women who were seeking health care; the rates of physical disease and mental illness in women who do not seek health care may be higher. Related to this point, the external validity of our results may be limited because the sociodemographic and clinical characteristics of Syrian refugee women living in camps or other countries vary considerably. Finally, our findings may not be used to compare the rates of miscarriage and modifiable risk factors related to miscarriage among Syrian refugee women to host communities because our analysis did not include vulnerable Jordanian women.

## Conclusions

We identified several risk factors for miscarriage in Syrian refugee women living in non-camp settings in Jordan. Our findings suggest that thyroid diseases may be poorly controlled in women who miscarried. Routine prenatal care for Syrian refugee women in Jordan should include a special emphasis on thyroid diseases; health professionals should actively seek to identify at-risk women and manage the disease in women with an established diagnosis. In addition, we identified a subset of women of advanced maternal age who did not want more children but did not use contraception. Miscarriages associated with advanced maternal age may be limited by increasing the access of these women to family planning services. All in all, the risk factors we identified may be amenable to preconception and prenatal care.

## Data Availability

The data that support the findings of this study are available from the corresponding author on reasonable request.

## Abbreviations

UNHCR: United Nations high commissioner for refugees
CSI: Coping strategies index
CES–D four-item scale: : Center for epidemiologic studies depression four-item scale
GAD–7 Scale: : Generalized anxiety disorder seven-item scale
PCL–5: : Posttraumatic stress disorder checklist for DSM–5
